# The Vaginal Microbiota of Adult Malagasy Women of Reproductive Age in the Marovoay District: First Characterization and Exploration of Associations With Human Papillomavirus and *Schistosoma haematobium* Infections

**DOI:** 10.1093/infdis/jiag065

**Published:** 2026-02-04

**Authors:** Jana Christina Hey, Johanna Saalfrank, Tahinamandranto Rasamoelina, Anjarasoa Ravo Razafindrakoto, Matthieu Razafindralava, Valeri Gildas Rajaoniarivo, Jean-Marc Kutz, Sonya Ratefiarisoa, Thorsten Thye, Irina Kislaya, Simon Graspeuntner, Zoly Rakotomalala, Bodo Sahondra Randrianasolo, Rivo Solotiana Rakotomalala, Valentina Marchese, Raphael Rakotozandrindrainy, Diavolana Koecher-Andrianarimanana, Pia Rausche, Aaron Remkes, Jürgen May, Pytsje T Hoekstra, Govert J van Dam, Paul L A M Corstjens, Tarik Gheit, Rivo Andry Rakotoarivelo, Corinna Bang, Daniela Fusco

**Affiliations:** Research Group Implementation Research, Bernhard Nocht Institute for Tropical Medicine (BNITM), Hamburg, Germany; German Center for Infection Research (DZIF), Hamburg-Borstel-Lübeck-Riems, Germany; Research Group Immunogenetics and Bioinformatics, Institute of Clinical Molecular Biology (IKMB), Christian-Albrechts-University of Kiel, Kiel, Germany; Research Group Implementation Research, Bernhard Nocht Institute for Tropical Medicine (BNITM), Hamburg, Germany; Centre d’Infectiologie Charles Mérieux (CICM), University of Antananarivo, Antananarivo, Madagascar; Centre d’Infectiologie Charles Mérieux (CICM), University of Antananarivo, Antananarivo, Madagascar; Centre d’Infectiologie Charles Mérieux (CICM), University of Antananarivo, Antananarivo, Madagascar; Mother-Child Complex, Centre Hospitalier Universitaire (CHU) Androva, Mahajanga, Madagascar; Research Group Implementation Research, Bernhard Nocht Institute for Tropical Medicine (BNITM), Hamburg, Germany; German Center for Infection Research (DZIF), Hamburg-Borstel-Lübeck-Riems, Germany; Mother-Child Complex, Centre Hospitalier Universitaire (CHU) Androva, Mahajanga, Madagascar; Department of Infectious Diseases Epidemiology, Bernhard Nocht Institute for Tropical Medicine (BNITM), Hamburg, Germany; Research Group Implementation Research, Bernhard Nocht Institute for Tropical Medicine (BNITM), Hamburg, Germany; German Center for Infection Research (DZIF), Hamburg-Borstel-Lübeck-Riems, Germany; Department of Infectious Diseases Epidemiology, Bernhard Nocht Institute for Tropical Medicine (BNITM), Hamburg, Germany; German Center for Infection Research (DZIF), Hamburg-Borstel-Lübeck-Riems, Germany; Medical Clinic III, University of Lübeck, Lübeck, Germany; Mother-Child Complex, Centre Hospitalier Universitaire (CHU) Androva, Mahajanga, Madagascar; Association K’OLO VANONA, Antananarivo, Madagascar; Mother-Child Complex, Centre Hospitalier Universitaire (CHU) Androva, Mahajanga, Madagascar; Research Group Implementation Research, Bernhard Nocht Institute for Tropical Medicine (BNITM), Hamburg, Germany; German Center for Infection Research (DZIF), Hamburg-Borstel-Lübeck-Riems, Germany; Department of Microbiology and Parasitology, University of Antananarivo, Antananarivo, Madagascar; Mother-Child Complex, Centre Hospitalier Universitaire (CHU) Androva, Mahajanga, Madagascar; Research Group Implementation Research, Bernhard Nocht Institute for Tropical Medicine (BNITM), Hamburg, Germany; German Center for Infection Research (DZIF), Hamburg-Borstel-Lübeck-Riems, Germany; Research Group Implementation Research, Bernhard Nocht Institute for Tropical Medicine (BNITM), Hamburg, Germany; German Center for Infection Research (DZIF), Hamburg-Borstel-Lübeck-Riems, Germany; German Center for Infection Research (DZIF), Hamburg-Borstel-Lübeck-Riems, Germany; Department of Infectious Diseases Epidemiology, Bernhard Nocht Institute for Tropical Medicine (BNITM), Hamburg, Germany; Tropical Medicine I, University Medical Center Hamburg-Eppendorf (UKE), Hamburg, Germany; Leiden University Center for Infectious Diseases, Leiden University Medical Center, Leiden, the Netherlands; Leiden University Center for Infectious Diseases, Leiden University Medical Center, Leiden, the Netherlands; Department of Cell and Chemical Biology, Leiden University Medical Center, Leiden, the Netherlands; Epigenomics and Mechanisms Branch, International Agency for Research on Cancer (IARC), Lyon, France; Research Group Implementation Research, Bernhard Nocht Institute for Tropical Medicine (BNITM), Hamburg, Germany; Department of Infectious Diseases, University of Fianarantsoa Andrainjato, Fianarantsoa, Madagascar; Research Group Immunogenetics and Bioinformatics, Institute of Clinical Molecular Biology (IKMB), Christian-Albrechts-University of Kiel, Kiel, Germany; Research Group Implementation Research, Bernhard Nocht Institute for Tropical Medicine (BNITM), Hamburg, Germany; German Center for Infection Research (DZIF), Hamburg-Borstel-Lübeck-Riems, Germany

**Keywords:** vaginal microbiota, human papillomavirus, *Schistosoma haematobium*, female genital schistosomiasis, Madagascar

## Abstract

**Background:**

The vaginal microbiome plays an important role for women's health. Changes in its composition have been associated with several sexually transmitted infections, including human papillomavirus (HPV) or parasitic infections such as *Schistosoma haematobium*. In Madagascar, gynecological conditions such as chronic manifestations of *S. haematobium* infections (female genital schistosomiasis), HPV infections, and cervical cancer are highly prevalent; however, data on the interplay between these conditions and the vaginal microbiota (VM) are still scarce. In addition, the majority of data originates from the Global North, generating a biased understanding of “healthy” VM across different geographic and social contexts. The objective of our study was to characterize for the first time the VM of adult women of reproductive age in Madagascar and to describe the variability of the vaginal environment in the presence of 3 conditions affecting the urogenital tract.

**Methods and Results:**

We characterized the VM of 443 participants, identifying the 5 community state types (CSTs I–V), with CST IV (57.1%, diverse) and CST III (34.1%, *Lactobacillus iners* dominated) as the most prevalent. CSTs were associated with previous antibiotics usage, while variability in the alpha and beta diversity was associated with dietary behavior and previous antibiotic usage. Differential abundance analysis showed variations among specific taxa in HPV-positive and female genital schistosomiasis–positive participants.

**Conclusions:**

With this first study of the VM in Madagascar we contribute to a broader understanding of vaginal health, as well as narrowing the gap of VM research in sub-Saharan Africa by enriching microbiota databases.

Women's health is a critical component of public health; however, it has been historically underrepresented in biomedical research and clinical trials [1]. In particular, conditions related to reproductive and gynecological well-being, such as sexually transmitted infections (STIs) and bacterial vaginosis, are among the most pressing issues women face [1, 2]. Among the most critical, yet understudied, contributor to reproductive health and gynecological conditions are the vaginal microbiota (VM), providing the first line of defense in the female genital tract [2, 3].

A healthy VM composition has long been characterized in the literature by the dominance of lactobacilli [4–7]. These bacteria produce lactic acid and other antimicrobial peptides, inhibiting the overgrowth of anaerobic bacteria and sexually transmitted pathogens [4, 5, 8]. A lack of lactobacilli in the VM composition has been associated with a high risk of gynecological conditions and reproductive diseases, such as the acquisition of STIs, preterm birth, miscarriages, and infertility [2, 8–10]. Until today, studies of VM have described 5 main vaginal environments in healthy and asymptomatic women: the so-called community state types (CSTs) [6]. Each CST is dominated by a specific *Lactobacillus* species: *L. crispatus*, *L. gasseri*, *L. iners*, and *L. jensenii* dominate CST I, II, III, and V, respectively; whereas CST IV is not dominated by lactobacilli but characterized by a higher diversity of anaerobic bacteria, such as *Gardnerella*, *Fannyhessea*, and *Prevotella* [6]. This CST has furthermore been linked to several gynecological conditions, such as bacterial vaginosis, human immunodeficiency virus, and human papillomavirus (HPV) infections [7, 11].

However, these CST findings have been primarily based on cohorts from the Global North [5, 6, 12], leading to an underrepresentation of vaginal microbiome research in low- and middle-income countries, especially in sub-Saharan Africa (SSA) [2]. Studies conducted so far in women with African or Latin American ancestries living in high-income countries, as well as those living in SSA [13–16], have described a lower proportion of lactobacilli-dominated VM and a higher prevalence of diverse VM than in white and Asian women [6, 12]. However, it remains to be determined whether these microbial differences can be explained by a more pathogenic or dysbiotic vaginal environment, associated with specific geographic and social contexts.

One of the SSA countries where research on the VM is completely absent is Madagascar [17], where gynecological conditions such as HPV infections (>39%), cervical cancer (CC), urogenital schistosomiasis, caused by an infection with *Schistosoma haematobium*, and its gynecological consequences (female genital schistosomiasis [FGS]) (>60%) are highly prevalent [18, 19]. CC is a high-burden disorder mainly caused by infection with HPV [20], which can be prevented through vaccination [21], but this is not yet implemented in Madagascar [22]. Persistent infections with *S. haematobium* can lead to FGS, and both have been suggested to increase the risk of CC [23, 24].

The body of literature showing the role of VM in the persistence of HPV infection, and consequently the onset of CC, is expanding [7, 25]. Specifically, microbiota lacking lactobacilli and with higher microbial diversity are described to produce fewer antimicrobial peptides and lactic acid, leading to prolonged inflammation and damage of the vaginal epithelial cells and thus easing the entry of viruses—such as HPV—into target tissues [7]. So far, few studies have described the characteristics and variability of the VM in association with *S. haematobium* infections or FGS [26, 27]. Overall, individuals carrying a high intensity of *S. haematobium* infection exhibit more diverse bacterial communities [26], and *Trichomonas vaginalis* seems to be more abundant in the presence of FGS [27]. Nevertheless, the association of *S. haematobium* infection and FGS with the VM composition remains to be defined.

Thus, the objective of our current study was to characterize for the very first time the VM of adult Malagasy women of reproductive age resident in the district of Marovoay and to describe differences and variability of the vaginal environment in the presence of 3 highly prevalent conditions affecting the urogenital tract—HPV infection, *S. haematobium* infection, and FGS.

## METHODS

### Study Design, Data, and Sample Collection

This is a secondary analysis of a cross-sectional study conducted at 3 primary healthcare centers in the *S. haematobium*–endemic area of the district of Marovoay, in the Boeny region of Madagascar, as described elsewhere [18]. Data were collected from 500 women aged 18–49 years between March and August 2021. Gynecological examinations were performed by midwives, including the capture of colposcopic images and the collection of cervicovaginal swab (CVS) and urine samples. Background characteristics of each participant were collected, including sociodemographics, personal habits, and clinical histories. CVS samples were directly eluted in ThinPrep tubes with 20 mL of PreservCyt solution and, together with urine samples, were stored according to standard conditions before shipment to Europe for analysis.

### Diagnostics of HPV Infection, *S. haematobium* Infection, and FGS

CVS eluates were analyzed at the International Agency for Research on Cancer in Lyon, France. There, a standardized Luminex-bead based assay (E7-MPG; International Agency for Research on Cancer) was performed to identify major high- and low-risk HPV types [18, 28, 29]. Samples were considered positive if ≥1 type was detected, regardless whether it was high, probable high, or low risk.

Active schistosome infections were diagnosed through the upconverting reporter particle, lateral flow test (UCP-LF) detecting the circulating anodic antigen (CAA) produced by schistosomes in urine samples, as described elsewhere [30]. Samples were considered positive if the circulating anodic antigen concentration exceeded the assay cutoff threshold for positivity of 2 pg/mL (UCAA*hT*417) [31].

FGS was diagnosed through the double-blinded assessment of the capture of colposcopic images after recruitment by 2 expert gynecologists, based on the 4 typical cervicovaginal signs described by the World Health Organization FGS atlas [32]. Images with a concordant rating were classified as either negative or positive and were included in the analysis. Discordant ratings were classified as indeterminate and were excluded from the analysis.

### 16S Ribosomal RNA Gene Amplicon Sequencing From CVS Eluates

The sequencing of CVS eluates for the microbiota analysis was performed at the Institute for Clinical Molecular Biology (IKMB) in Kiel, Germany.

#### DNA Extraction

DNA was extracted following the spin protocol from the QIAamp UCP Pathogen Mini Kit (Qiagen), according to the manufacturer's instructions. The samples with bead beating were pretreated to allow proper lysis of membranes by adding 500 μL of the respective sample to a Pathogen Lysis Tube S (Qiagen), containing small silica beads as supplied by the manufacturer and 200 μL of ATL Buffer (Qiagen). After incubation for 10 minutes at 56°C and 500 rpm, samples were shaken in a speed mill for 45 seconds, and the tubes were centrifuged for 1:30 minutes at 15 000 rpm. Samples were stored at −20°C until further use.

#### Amplification of the V3/V4 Variable Region of the 16S Ribosomal RNA Gene

The V3/V4 variable regions of the 16S ribosomal RNA (rRNA) gene were amplified in a 1-step polymerase chain reaction (PCR) on 96-well plates using the primer pair 341F-806R (dual-barcoding approach; primer sequences 5'-CCTACGGGAGGCAGCAG-3’ and 5'-GGACTACHVGGGTWTCTAAT-3') [33], as described elsewhere [34] (Supplementary Table 1). PCR products were stored at −20°C after verification of their presence by capillary gel electrophoresis. Nontemplate controls (RNA/DNA-free water) and mock community controls (ZymoBIOMICS Microbial Community DNA Standard; Zymo Research) were added to every plate (results reported in Supplementary Figures 1 and 2).

#### Normalization Assay

To purify the PCR products and ensure an equal amount of each product for the sequencing pool, a normalization assay was conducted using the SequalPrep Normalization Plate Kit (Thermo Fisher Scientific). The Qubit 2.0 Fluorometer (Thermo Fischer Scientific) was used to quantify the DNA by plate using the double-stranded DNA “Broad Range” program. Depending on the quantity of DNA, eluates were pooled equimolarly, each pool containing a maximum of 288 different samples. Pools were stored at −20°C until sequencing.

### rRNA Gene Amplicon Sequencing

The 16S rRNA gene amplicon sequencing was performed using paired-end MiSeq sequencing (Illumina) with a reading length of 2 × 300 base pairs and conducted on the MiSeq platform (MiSeqFGx; Illumina), using the MiSeq Reagent Kit v3. The settings for demultiplexing were 0 mismatches in the barcode sequences. Quality control, trimming, and filtering of the sequencing files were performed using an established pipeline at IKMB [35]. Merged reads were assigned to the SILVA 138.2 database. To overcome the limitations of V3/V4 16S sequencing to identify bacterial taxa down to the species level, the speciateIT classification tool was used. Taxonomies with a probability >80% were included in the taxonomic annotation.

### Statistical Analysis

Characteristics of the study participants were described in terms of absolute and relative frequencies for categorical variables, and median and interquartile range (IQR) for numerical variables. We used the prevalence-method of the R package “decontam” to assess contamination and decontaminate the sequencing data prior to further analysis (Supplementary Table 2). The composition of the VM was described in terms of alpha and beta diversity as well as relative abundance. Alpha diversity analysis was conducted using the R package “vegan,” based on the Shannon diversity index. A multivariate linear regression was conducted, and estimates were displayed using forest plot together with 95% confidence intervals (CI) and *P* values. Beta diversity was assessed using Bray-Curtis dissimilarity metrics. To estimate associations between beta diversity and participant characteristics, the Adonis test from the R package “vegan” was performed. Relative abundances were computed using the R package “microbiome.” Using the aggregate_rare function of the package with 1% prevalence and 1% detection as the thresholds, bacterial species were displayed in a heat map, using the R package “pheatmap.”

To identify CSTs, samples were mapped to the VALENCIA CSTs by manually matching taxa between the SILVA 138.2/speciateIT taxonomy and the VALENCIA taxonomy [36]. To assess associations between categorical variables, χ^2^ or Fisher exact tests were used.

Analysis of differential abundance of taxa between groups was performed using the Analysis of Compositions of Microbiomes with Bias Correction 2 (ANCOM-BC2) method and the R package “ANCOMBC.” To reduce background noise and minimize the influence of potential contaminants, taxa present in <10% of samples and a read count <20 reads per sample were excluded. Multiple testing correction was done using the Holm procedure.

All analyses were conducted using R software, version 4.4.2. A complete list of R packages and pipelines from code repositories used is available in the Supplementary Methods.

### Ethical Considerations

Ethical approval was obtained from the National Ethics Committee of Madagascar (reference no. 052-MSANP/SG/AMM/CERBM) and the Ethics Committee Hamburg State Medical Chamber, Germany (reference no. PV7309). All participants were informed of the aims and procedures of the study in the Malagasy language. Participation in the study was voluntary. Informed consent was obtained from the participant by signature or, in the case of illiteracy, by thumbprint in the presence of an independent witness. Participants had the right to refuse to participate and to withdraw informed consent at any time without giving reasons. No financial incentives were given. Diagnosed pathological conditions were treated according to national guidelines and free of charge.

## RESULTS

### Participant Characteristics

**Figure 1.**
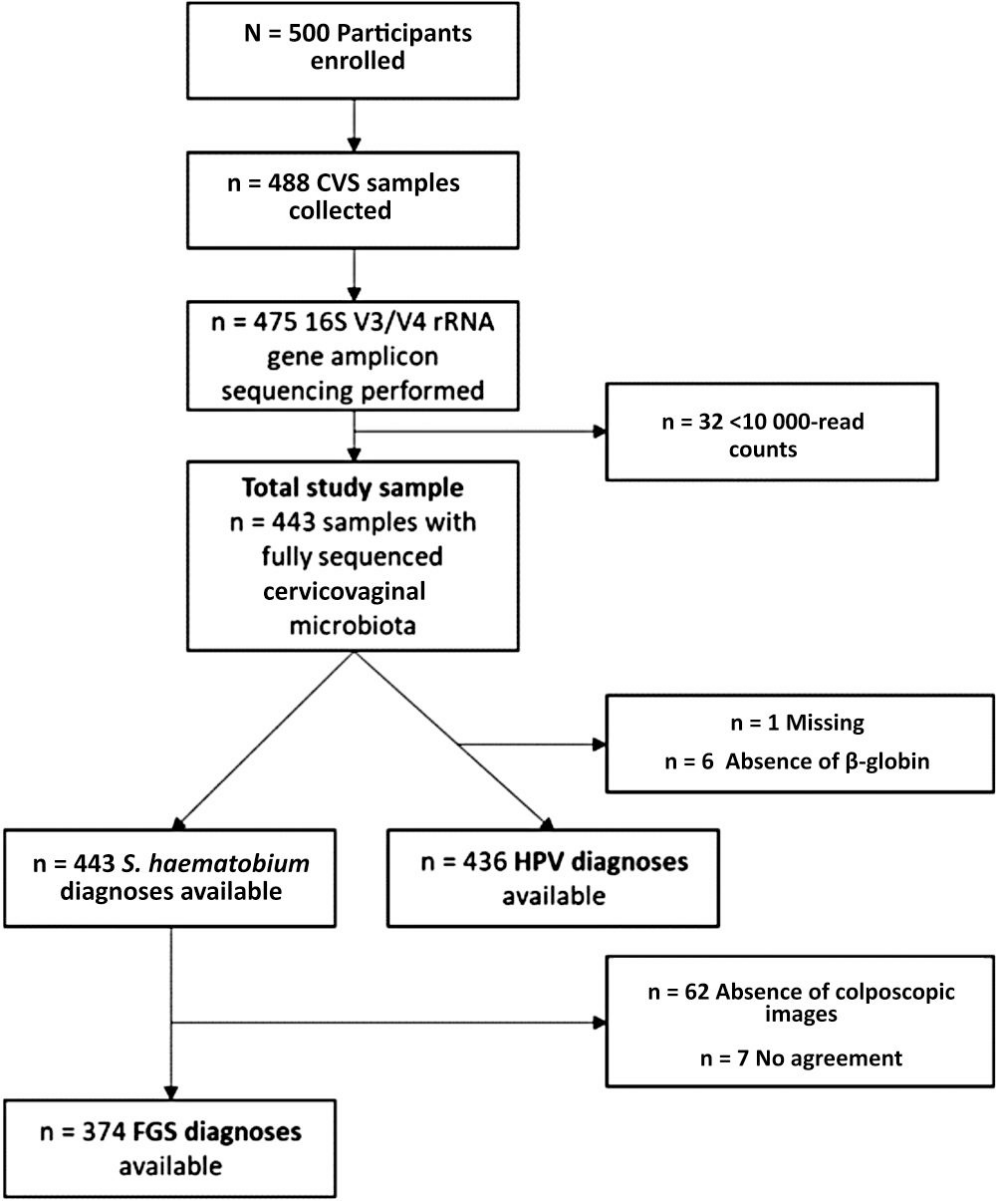
Inclusion flowchart. A breakdown of participant numbers in the 3 condition groups (*Schistosoma haematobium* infection, human papillomavirus [HPV] infection, and female genital schistosomiasis [FGS]) is available in Supplementary Table 3. Abbreviations: CVS, cervicovaginal swab; rRNA, ribosomal RNA.

A total of 500 participants were enrolled in the study. Of those, 443 could be included in the analysis (Figure 1). Participant characteristics are provided in Table 1. Of all participants, 45.0% (n = 196) were infected with HPV and 62.1% (n = 275) with *S. haematobium*, while 55.9% (n = 209) were diagnosed with FGS.

**Table 1.** Background Characteristics of Study Participants

Characteristic	Participants, No. (%)^a^ (n = 443)
Sociodemographics	
Age, median (IQR), y	30 (24–37)
Age group	
18–24 y	120 (27.1)
25–34 y	167 (37.7)
35–49 y	156 (35.2)
Urbanicity	
Rural	245 (55.3)
Periurban	198 (44.7)
Profession	
Nonfarmer	196 (44.2)
Farmer	247 (55.8)
Personal habits	
Smoking^b^	
No	421 (95.0)
Yes	22 (5.0)
Alcohol consumption^b^	
No	327 (73.8)
Yes	116 (26.2)
Meat consumption	
Never	98 (22.1)
1 d/wk	174 (39.3)
2 d/wk	112 (25.3)
≥3 d/wk	59 (13.3)
Fish consumption	
Never or 1 d/wk	17 (3.8)
2 d/wk	54 (12.2)
≥3 d/wk	372 (84.0)
Vegetable consumption	
Never	52 (11.7)
1 d/wk	75 (16.9)
2 d/wk	190 (42.9)
≥3 d/wk	126 (28.4)
Clinical history	
Previous treatment with PZQ^c,d^	
No	295 (66.6)
Yes	148 (33.4)
Previous use of antibiotics^d,e^	
No	377 (85.1)
Yes	66 (14.9)
Bleeding^d^	
No	343 (77.4)
Yes	100 (22.6)
Vaginal discharge^d^	
No	282 (63.7)
Yes	161 (36.3)
Itching^d^	
No	311 (70.2)
Yes	132 (29.8)
Pain during/after sexual intercourse^d^	
No	299 (67.5)
Yes	144 (32.5)
Previous pregnancies	
None	51 (11.5)
1	77 (17.4)
2	79 (17.8)
≥3	236 (53.3)
Previous miscarriage	
No	250 (56.4)
Yes	193 (43.6)
HPV infection (n = 436)	
Negative	240 (55.0)
Positive	196 (45.0)
HPV risk type (n = 436)	
No infection	240 (55.0)
Low risk/probable high risk	36 (8.3)
High risk	160 (36.7)
*Schistosoma haematobium* infection	
Negative	168 (37.9)
Positive	275 (62.1)
FGS diagnosis (n = 374)	
Negative	165 (44.1)
Positive	209 (55.9)
CST	
CST I-A	18 (4.1)
CST I-B	17 (3.8)
CST II	3 (0.7)
CST III-A	70 (15.8)
CST III-B	81 (18.3)
CST IV-A	43 (9.7)
CST IV-B	164 (37.0)
CST IV-C0	14 (3.2)
CST IV-C1	18 (4.1)
CST IV-C4	14 (3.2)
CST V	1 (0.2)

Abbreviations: CST, community state type; FGS, female genital schistosomiasis; HPV, human papillomavirus; IQR, interquartile range; PZQ, praziquantel.

^a^Data represent no. (%) of participants unless otherwise specified.

^b^“Yes” includes regular consumption and consumption on special occasions.

^c^“Yes” includes treatment within the past year or earlier.

^d^“No” includes “I don’t know.”

^e^Within the past month.

### Composition and Diversity of the VM

**Figure 2.**
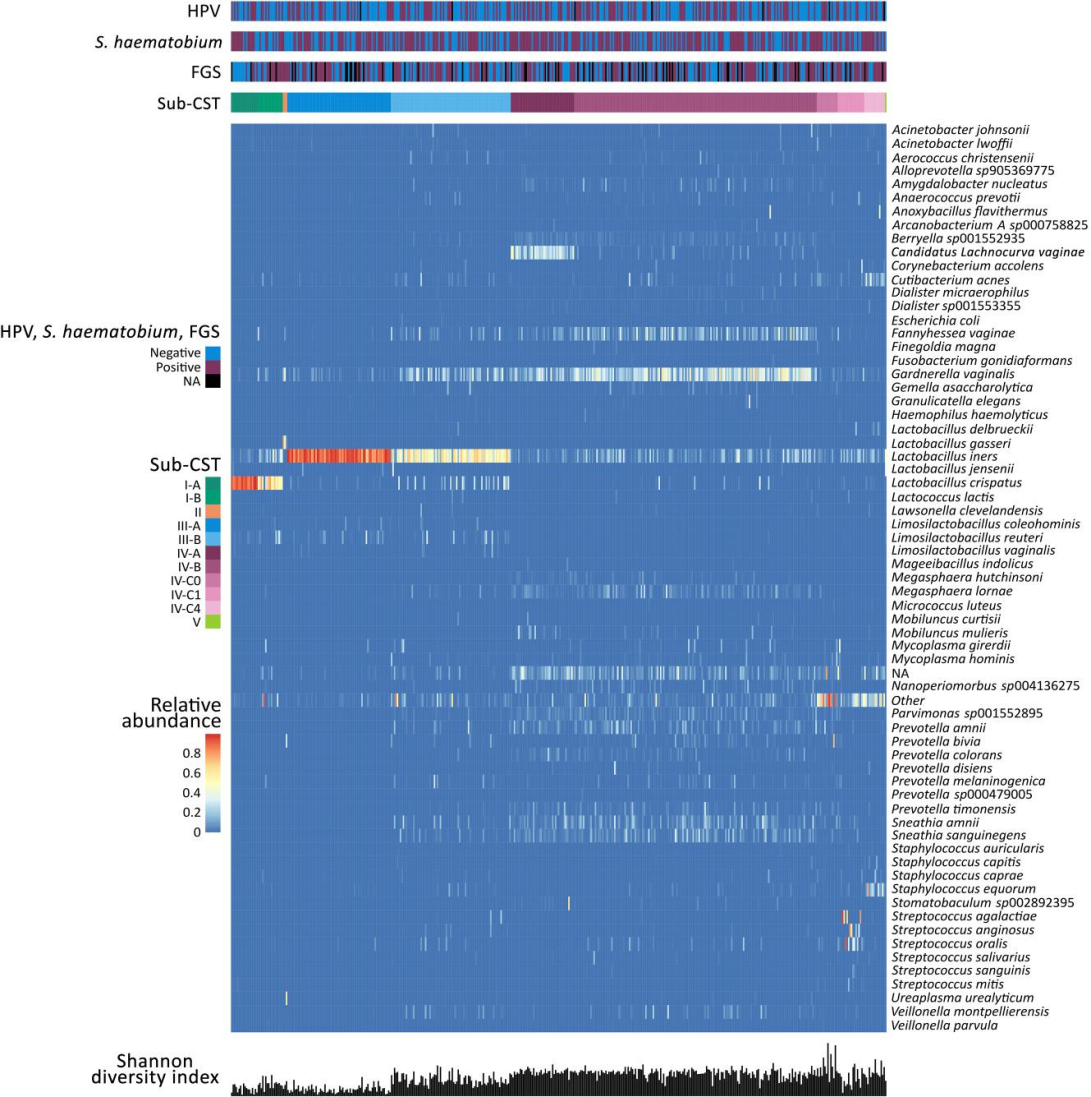
Overview of the vaginal bacterial communities identified in 443 adult Malagasy women of reproductive age. The heat map displays the relative abundance (with 1% prevalence and 1% detection thresholds) of key microbial species of all participants, ordered by subgroups of community state types (sub-CSTs). The human papillomavirus (HPV) and *Schistosoma haematobium* infection status as well as the female genital schistosomiasis (FGS) diagnosis are displayed above the heat map. The Shannon diversity indices for all samples are indicated below the heat map. Abbreviation: NA, not applicable.

The vaginal bacterial taxa of the 443 participants included in the study were grouped according to the VALENCIA community compositions. Overall, all 5 CSTs were identified in the study sample (Table 1), with the majority of VM classified as CST IV (57.1% [n = 253]), followed by CST III (34.1% [n = 151]) and CST I (7.9% [n = 35]). For CST I, III, and IV, sub-CSTs were further classified. The majority of study participants (37.0% [n = 164]) were grouped as CST IV-B. Only 0.7% (n = 3) and 0.2% (n = 1) were classified as CST II and V, respectively. The heat map in Figure 2 shows the relative abundance of bacterial species ordered by the sub-CSTs, highlighting the dominance of different lactobacilli species in CST I, II, and III as well as the high microbial diversity of CST IV, which is similarly reflected in the Shannon diversity index.

### Association of Sociodemographics, Personal Habits, and Clinical Histories With VM Composition

**Figure 3.**
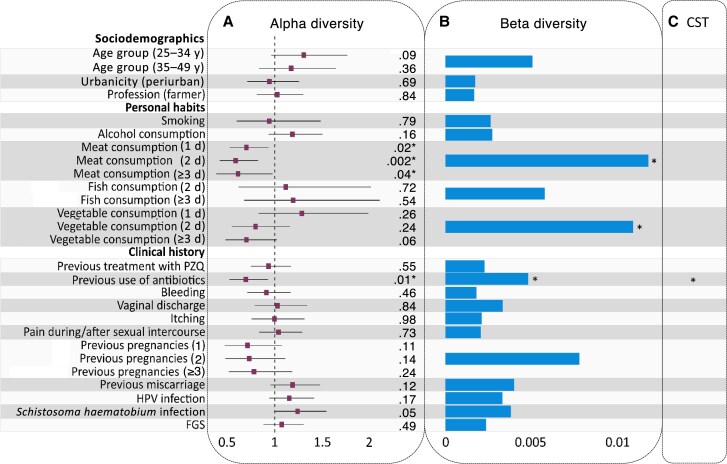
Analysis of the association of participant characteristics with vaginal microbiota composition. Each panel displays the association on a different level. *A,* Effect on alpha diversity (Shannon diversity index); multivariate linear regression model is adjusted for all variables, and the center is the effect size, with error bars displaying 95% confidence intervals and with *P* values provided on the right. *B,* Effect on beta diversity (Adonis test); model is adjusted for all variables, and bars represent *R*^2^ values, with significant *P* values displayed as asterisks. *C,* Association with community state type (CST) abundance (CST I-A, I-B, III-A, III-B, IV-A, IV-B, IV-C1; CSTs with <15 samples per group were excluded, and significant *P* values are displayed as asterisks. Abbreviations: FGS, female genital schistosomiasis; HPV, human papillomavirus; PZQ, praziquantel.

We then analyzed the association of participant characteristics with VM composition in terms of (1) alpha diversity, (2) beta diversity, and (3) CSTs. Alpha diversity was associated with dietary behavior and the previous use of antibiotics (Figure 3*A*). Regularly consuming meat (1 day per week, *P* = .02; 2 days, *P* = .002; ≥3 days, *P* = .04) as well as the previous use of antibiotics in the last month (*P* = .01) significantly reduced alpha diversity. Further trends in the same direction were visible for the regular consumption of vegetables (≥3 days per week, *P* = .06), whereas *S. haematobium* infection (*P* = .05) and age group (age 25–34 years, *P* = .09) seemingly increased alpha diversity. Besides dietary behavior (consumption of meat, *R*^2^ = 0.012 and *P* = .02; consumption of vegetables, *R*^2^ = 0.011 and *P* = .05) and the previous use of antibiotics (*R*^2^ = 0.005; *P* = .03) showing the strongest association with beta diversity, there was a trend toward a significant association with *S. haematobium* infection (*R*^2^ = 0.004; *P* = .08) and previous miscarriage (*R*^2^ = 0.004; *P* = .09) (Figure 3*B*).

CST abundance was associated with the previous use of antibiotics (*P* = .03) (Figure 3*C*). Among participants who reported the use of antibiotics in the month before enrollment, CST III was the most commonly observed (46.9%), followed by CTS IV (39.4%), while among participants who did not report antibiotic use, CST IV emerged as the predominant type, detected in 60.2% of participants. HPV infections (as well as HPV risk types [Supplementary Table 4]) and FGS positivity were not on any of the 3 levels associated with the VM composition (Figure 2 and Figure 3).

### Association of HPV, *S. haematobium*, and FGS With VM Composition

**Figure 4.**
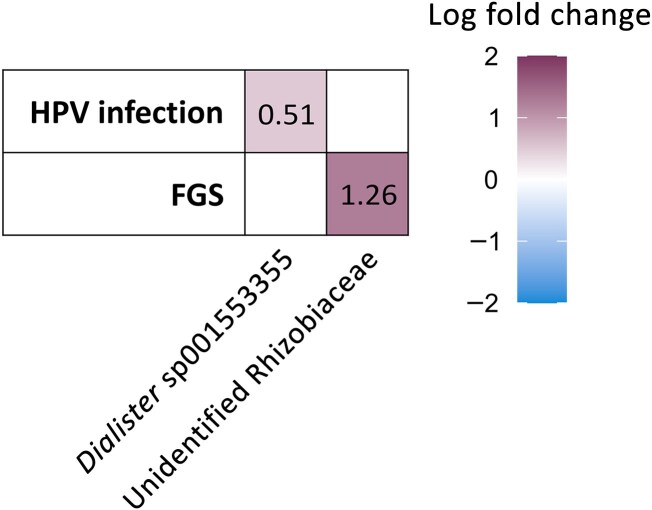
Differential abundance testing with the Analysis of Compositions of Microbiomes with Bias Correction 2 (ANCOM-BC2) method for different human papillomavirus (HPV) infection and female genital schistosomiasis (FGS) positivity statuses. Log fold changes of bacterial species are compared between HPV-positive and HPV-negative participants (HPV infection) and between FGS-positive and FGS-negative participants (FGS). Only significant results that passed the sensitivity analysis were displayed, and the output was adjusted for all available variables.

Differential abundance analysis using ANCOM-BC2 was performed among participants with HPV or *S. haematobium* infection or affected by FGS (Figure 4). Among HPV-positive individuals, only 1 differentially abundant taxon was observed: the species *Dialister* sp001553355 was significantly more abundant in HPV-infected than in HPV-negative participants. An unidentified species of the family Rhizobiaceae was significantly more abundant in FGS-positive than in FGS-negative individuals. The analysis did not identify any taxa with significant differential abundance among *S. haematobium*–infected individuals.

## DISCUSSION

The analysis of the VM described in this study is, to our best knowledge, the very first reporting the overall microbiota composition and its variability in the presence of highly prevalent conditions affecting the urogenital tract among adult women of reproductive age in the district of Marovoay in Madagascar.

We identified the 5 CSTs first described by Ravel et al in 2011 [6], with the majority of VM being categorized as CST IV, followed by CST III—which is in line with other studies from SSA. Compared with white or Asian women, in whom lactobacilli dominance is frequently described (mainly CST I and CST II) [5, 6, 12], more CST III and CST IV have been described in SSA populations [13–16, 37]. CST III is often considered to be a transitional/less stable CST, since *L. iners* produces only the less potent L-lactic acid, making this CST also more vulnerable to transitions to highly diverse or dysbiotic environments (CST IV) [8].

A high abundance of lactobacilli in the vaginal environment is normally considered a sign of eubiosis [4, 7, 8]. Since we did not identify a lactobacilli dominance, we can speculate that the population studied, as those from other SSA countries, has an overall VM composition generating a default vaginal environment that could be defined as dysbiotic. This may suggest that, in addition to human behavior, environmental and genetic factors can play a role in influencing the bacterial composition of the VM and that more diverse VM may not necessarily be indicative of disease [2, 6, 38, 39]. Taken together, our data show the importance of producing descriptive data on VM from different populations ultimately to better understand the role of specific microbial communities in vaginal health and gynecological conditions.

Furthermore, we observed a significant difference across microbial communities in the presence of HPV infection. Several studies shown have that in women infected with HPV a shift in the relative abundance occurs, determined by an increased representation of those highly diverse CSTs not dominated by lactobacilli [7, 16]. We identified a higher abundance of a *Dialister* species in HPV-infected individuals, which has been previously reported among the species prevalent in highly diverse VM associated with vaginal dysbiosis [5, 6]. In addition, increased levels of *Dialister* have been observed in HPV-related lesions and in the VM of women with CC [40, 41]. However, we were unable to observe a lower abundance of lactobacilli among HPV-positive participants. As our study sample was already highly diverse and generally dominated by anaerobic bacteria, this could have had an influence. The generally lower abundance of lactobacilli in combination with a high prevalence of highly diverse VM in our study sample could have consequences for vaginal health and HPV susceptibility.

We observed a significant difference in the VM in the presence of FGS. So far, few studies have investigated the variability of VM in the presence of *S. haematobium* or FGS, generally describing a more diverse bacterial community in the presence of the parasite [26, 27]. Even though our study sample had both (1) a higher proportion of highly variable microbiota and (2) a higher prevalence of *S. haematobium* and FGS in the area, we confirmed the trend of higher alpha diversity in our study sample and described a differentially abundant taxon (higher abundance of an unidentified species of the family Rhizobiaceae) in FGS-positive individuals. Further studies on longitudinal cohorts might help improve the understanding of HPV acquisition in FGS-affected women, while also determining the timing of a switch with the potential of identifying prognostic indicators for the occurrence of FGS after *S. haematobium* infection. Further investigations are needed in order to understand the association, implications, and mechanistic roles of these differentially abundant taxa in the vaginal environment, the disease progression of *S. haematobium* infections and FGS, and their influence on other gynecological conditions.

Despite the strengths and original findings of the present study, it does not come without limitations. The lack of detailed gynecological investigations of the study's participants did not allow us to draw clinical conclusions on the eubiotic versus dysbiotic vaginal environment of our study sample. Due to the sampling, sequencing, and analysis methods adopted, several challenges were encountered. First, the low taxonomic resolution left several sequences without species annotation [42]. Second, VALENCIA enabled reproducible and standardized CST classifications consistent with prior studies. However, a subset of samples (n = 16; mostly CST IV) received low-confidence scores (<0.1). These samples shed light on the fact that CST definitions are warranting optimization and revision as we learn more about rare datasets from understudied populations, such as the one presented here. Third, although the microbial diversity of some associations reached statistical significance (*P* < .05), the corresponding rather small effect sizes reflect the overall low-diversity structure of the VM, and interpretations require caution.

Fourth, contamination with bacterial DNA is always an issue with low-biomass microbiota, which we excluded as well as possible by reporting taxa found in nontemplate controls and applying several filtering methods. Nevertheless, the identified differentially abundant taxa should be interpreted with caution, as their presence may very well reflect true biological variation [43], but we cannot rule out the possibility of contamination that could have occurred, for example, during sample collection. At the same time, the filtering could have reduced the possibility of identifying important low-abundant taxa, which are often present in the vaginal environment and could play an additional role in HPV and *S. haematobium* infections or FGS. Given the high prevalence of the 3 conditions and the small number of participants completely free from any of the 3, our data were not compared with a “healthy” group. Finally, the secondary analysis and the cross-sectional nature of our study limited the ability to address the implications of the dynamic nature of the VM. Information about the menstrual cycle, hormonal contraception, the status of menopause, and other STIs was not available.

In conclusion, with our study we contribute to a broader understanding of vaginal health as well as narrowing the gap of VM research in SSA by enriching microbiota databases. This study further highlights the need for more studies from different regions of the globe to dissect the different effects of microbial communities on vaginal health. Furthermore, identifying elements influencing the variability of the VM for prevention, diagnosis, prognosis, and treatment of high-burden gynecological diseases has a high potential that deserves more attention and research from the biomedical and global health communities.

## Supplementary Material

jiag065_Supplementary_Data

## Supplementary Data

Supplementary materials are available at *The Journal of Infectious Diseases* online (http://jid.oxfordjournals.org/). Supplementary materials consist of data provided by the author that are published to benefit the reader. The posted materials are not copyedited. The contents of all supplementary data are the sole responsibility of the authors. Questions or messages regarding errors should be addressed to the author.
