# Supplementary material for: The Vaginal Microbiota of Adult Malagasy Women of Reproductive Age in the Marovoay District: First Characterization and Exploration of Associations With Human Papillomavirus and Schistosoma haematobium Infections
Source: J Infect Dis. 2026 Feb 4;234(1):e90–e100. doi: 10.1093/infdis/jiag065 (PMC13431661; doi:10.1093/infdis/jiag065)
Supplement: jiag065_Supplementary_Data [file jiag065_supplementary_data.docx]

**Supplementary Materials**

1. **Supplementary Methods**

Ravel-Lab. speciateIT. 2025. Available at: <https://github.com/ravel-lab/speciateIT>. Accessed 6 August 2025.

Davis NM, Proctor DM, Holmes SP, et al. Simple statistical identification and removal of contaminant sequences in marker-gene and metagenomics data. *Microbiome* 2018, 6, 226.

Oksanen J, Simpson GL, Blanchet FG, et al*.* vegan: Community Ecology Package. 2022. Available at: <https://cran.r-project.org/web/packages/vegan/index.html>. Accessed 6 August 2025.

Lahti L. microbiome. 2025. Available at: <https://github.com/microbiome/microbiome>. Accessed 6 August 2025.

Kolde R. pheatmap: Pretty Heatmaps. R package version 1.0.13. 2025. Available at: <https://github.com/raivokolde/pheatmap>. Accessed 6 August 2025.

Ravel-Lab. VALENCIA. 2025. Available at: <https://github.com/ravel-lab/VALENCIA>. Accessed 6 August 2025.

Lin H, Peddada SD. Analysis of compositions of microbiomes with bias correction. *Nat Commun* 2020;11:3514.

R Core Team. R: A Language and Environment for Statistical Computing. 2024. Available at: <https://www.R-project.org/>. Accessed 6 August 2025.

1. **Supplementary Figures**

**
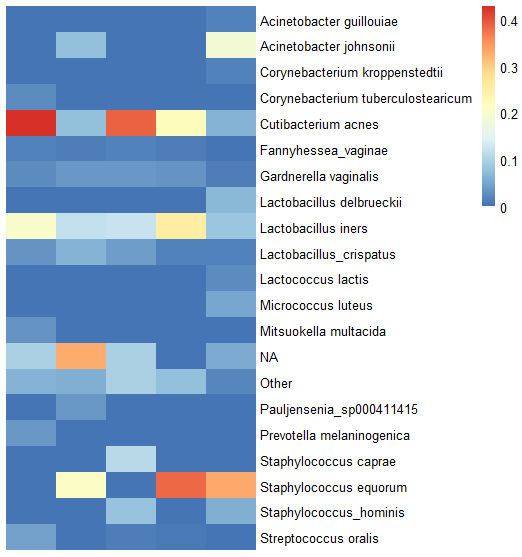
**

**Supplementary Figure 1:** Relative abundance heatmap for bacterial taxa present in five NTCs after removal of taxa given in Supplementary Table 2 (with 1 % prevalence and 1 % detection thresholds).

**
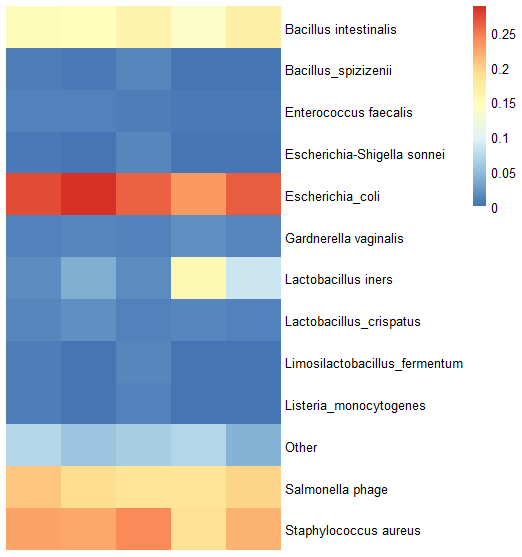
**

**Supplementary Figure 2**: Relative abundance heatmap for bacterial taxa in five MOCKs after removal of taxa given in Supplementary Table 2 (with 1 % prevalence and 1 % detection thresholds).

1. **Supplementary Tables**

**Supplementary Table 1:** Additional information for PCR amplification.

| **PCR step** | **Temperature** | **Time** | **Notes** |
| --- | --- | --- | --- |
| Initial denaturation | 98 °C | 30 seconds |  |
| Denaturation | 98 °C | 9 seconds | Amplification cycle for 30 cycles |
| Annealing | 55 °C | 1 minute |  |
| Extension | 72 °C | 45 seconds |  |
| Final extension | 72 °C | 10 minutes |  |

**Supplementary Table 2:** Overview sequences identified as contaminants based on decontam algorithm with prevalence-method.

| **Removed contaminants** |
| --- |
| Prevotella bivia |
| Bifidobacterium breve |
| Paracoccus NA |
| Gemella_sanguinis |
| Sphingomonadaceae NA NA |
| Prevotella_copri_B |
| Microbacterium oleivorans |
| Sphingomonas NA |
| Peptostreptococcus_sp000758885 |
| Ruminococcus NA |
| Streptococcus parasanguinis |
| Chloroplast NA NA NA |
| Hoylesella NA |
| Staphylococcus epidermidis |
| Schaalia odontolytica |
| Flavobacterium NA |
| Haemophilus haemolyticus |
| Flavobacterium limicola |
| Corynebacterium otitidis |
| Escherichia-Shigella coli |
| Massilia putida |
| Corynebacterium accolens |
| [Eubacterium] coprostanoligenes group NA NA |
| 67-14 NA NA |
| UCG-001 NA |
| Enhydrobacter NA |
| Blastocatellia NA NA NA NA |
| Microbacterium NA |
| Chryseobacterium hominis |
| Rothia mucilaginosa |
| Flavobacterium resistens |
| CENA359 NA |
| Dankookia rubra |
| Pseudomonas corrugata |
| Prevotella histicola |
| Anaerococcus NA |
| **Removed contaminants** |
| Muribaculum NA |
| Chloroplast NA NA NA |
| Pauljensenia_sp902373545 |
| Acinetobacter johnsonii |
| Streptococcus salivarius |
| Candidatus Alysiosphaera NA |
| Xanthomonas citri |
| Limosilactobacillus_fermentum |
| Comamonas aquatica |
| Lachnospiraceae NK4A136 group NA |
| Bacillus halotolerans |
| Blastocatellia NA NA NA NA |
| Muribaculaceae NA NA |
| Listeria monocytogenes |
| Hoylesella NA |
| Staphylococcus aureus |
| Salmonella enterica |
| Candidatus Alysiosphaera NA |
| Lawsonella clevelandensis |
| Mucilaginibacter NA |
| Stenotrophomonas nitritireducens |
| Corynebacterium tuberculostearicum |
| Pseudomonas aeruginosa |
| Prevotellaceae NK3B31 group NA |
| Enterococcus faecalis |

**Supplementary Table 3**: Breakdown of the participant numbers in the three condition groups (Sh infection, HPV infection, FGS).

| Sh diagnosis available for n = 443 | | | | | | | |
| --- | --- | --- | --- | --- | --- | --- | --- |
| Sh+  n = 275 | | | | Sh-  n = 168 | | | |
| HPV diagnosis available for n = 436  (no HPV analysis in n = 1, no beta globin control in n = 6) | | | | | | | |
| HPV+  n = 116 | | HPV-  n = 154 | | HPV+  n = 80 | | HPV-  n = 86 | |
| FGS diagnosis in n = 374  (**only n =** **369** **have a diagnosis of Sh, HPV, and FGS**) | | | | | | | |
| FGS+  n = 56 | FGS-  n = 41 | FGS+  n = 73 | FGS-  n = 57 | FGS+  n = 29 | FGS-  n = 33 | FGS+  n = 47 | **FGS-**  **n = 33** |

**Supplementary Table 4:** Results for association of HPV risk types with the VM composition.

| **Alpha diversity** | | | | | | | | |
| --- | --- | --- | --- | --- | --- | --- | --- | --- |
| **Shannon diversity index*** | | | **Adjusted estimate** | | **95 % CI** | | **p-value** | |
| Low-risk/probable high-risk | | | 1.281 | | 0.859-1.912 | | 0.224 | |
| High-risk | | | 1.130 | | 0.913-1.398 | | 0.260 | |
| **Beta diversity** | | | | | | | | |
| **Beta diversity** | | | **R^2^** | | | **p-value** | | |
| HPV risk types | | | 0.00532 | | | 0.435 | | |
| **CST association** | | | | | | | | |
| n (%) | **CST I-A** | **CST I-B** | **CST III-A** | **CST III-B** | **CST IV-A** | **CST IV-B** | **CST IV-C1** | **p-value** |
| No infection | 9 (2.22) | 10 (2.46) | 41 (10.10) | 44 (10.84) | 21 (5.17) | 85 (20.94) | 12 (2.96) | 0.763 |
| Low-risk/probable high-risk | 1 (0.25) | 1 (0.25) | 2 (0.49) | 11 (2.71) | 4 (0.99) | 15 (3.69) | 1 (0.25) |  |
| High-risk | 8 (1.97) | 6 (1.48) | 26 (6.40) | 25 (6.16) | 18 (4.43) | 61 (15.02) | 5 (1.23) |  |

* Reference category: No infection
